# Botulinum Toxin Type A in the Treatment of Primary Axillary Hyperhidrosis: A Phase 3, Multicenter, Randomized, Double-Blind, Placebo-Controlled Study of Efficacy and Safety in Chinese Patients

**DOI:** 10.1093/asj/sjaf260

**Published:** 2026-01-19

**Authors:** Yiming Li, Baogang Sun, Jianyun Lu, Wei Lai, Huiping Wang, Qiuning Sun, Baoxi Wang, Yanyan Feng, Junfeng He, Yubao Huang, Yunkai Yang, Chengjun Zhang, Li Li

## Abstract

**Background:**

The efficacy and safety of botulinum toxin type A (BoNTA) treatment for primary axillary hyperhidrosis (PAH) have not been explored in the Chinese population.

**Objectives:**

The objective was to evaluate efficacy and safety of 1 intradermal BoNTA injection in Chinese PAH cases.

**Methods:**

This was a Phase 3, multicenter, randomized, double-blind, placebo-controlled study. Patients were randomized to an experimental group or the control group at a ratio of 3:1 and received either BoNTA or a placebo once. The primary efficacy endpoint was the proportion of patients who experienced an over 50% reduction in axillary sweat weight at Week 4 posttreatment compared to baseline. The key secondary efficacy endpoints were the percentage changes in axillary sweat weight at Weeks 1, 4, 8, and 16 posttreatment.

**Results:**

A total of 344 patients were randomized to the experimental group (*n* = 258) or the control group (*n* = 86). The proportions of patients who experienced an over 50% reduction in axillary sweat weight at Week 4 posttreatment were 83.72% (216/258) in the experimental group and 55.81% (48/86) in the control group, respectively. The between-group difference was 27.91% (*P* < .001). BoNTA treatment yielded a significant reduction in axillary sweat weight, hyperhidrotic area, hyperhidrosis disease severity scale (HDSS) scores, and grade of bromhidrosis. The patients in the experimental group reported significantly higher satisfaction scores than those in the control group. BoNTA treatment was well tolerated. Neither group experienced suspected unexpected serious adverse reactions, or adverse events or adverse drug reactions leading to withdrawal or death.

**Conclusions:**

One intradermal 50-U BoNTA treatment led to a significant reduction in axillary sweat weight, axillary hyperhidrotic area, HDSS scores, and axillary bromhidrosis grades in Chinese PAH patients. The therapeutic effect was maintained for 16 weeks posttreatment, with a favorable safety profile.

**Level of Evidence: 1 (Therapeutic):**

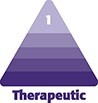

Hyperhidrosis (HH) is characterized by excessive sweating that goes beyond the needs of thermoregulation and significantly impairs patients' quality of life. Primary hyperhidrosis (PHH) is idiopathic and accounts for the majority of HH cases, which bilaterally affects the axillae, palms, soles, or craniofacial regions.^1-4^ Based upon a survey conducted in patients attending outpatient clinics in a Shanghai skin disease hospital, the prevalence of PHH in the Chinese population is 14.5%.^5^

Surgical and nonsurgical therapeutic modalities have been established, including topical agents, systemic medications, iontophoresis, botulinum toxin injections, energy devices, fractionated microneedle radiofrequency, and endoscopic sympathectomy.^1,3,6^ Topical antiperspirants are a first-line, nonsurgical option, but they lack long-term effectiveness, require repetitive applications, and can lead to secondary dermal conditions. Surgical measures bear the risk for wound infections and scars, and require anesthesia.^4^ These limitations highlight the necessity for a long-acting treatment option with a favorable safety profile.

Studies have established that treatment of primary axillary hyperhidrosis (PAH) with botulinum toxin type A (BoNTA) injection is safe and efficient.^7-9^ Although BoNTA inhibits skeletal muscular contractions for approximately 3 to 4 months, inhibition of autonomic cholinergic nerve terminals may last for 6 to 8 months.^1^ Satisfaction with BoNTA treatment proved to be significantly higher than other nonsurgical methods, even if it is recommended as a second-line treatment, once topical strategies have failed.^1,3,6^

The safety and efficacy of BoNTA treatment for PAH have not been studied in the Chinese population. Accordingly, we conducted a Phase 3, multicenter, randomized, double-blind, placebo-controlled study to investigate the aforementioned issues.

## METHOD

### Study Design

This was a Phase 3, multicenter, randomized, double-blind, placebo-controlled study (https://www.chictr.org.cn identifier: ChiCTR2500104529) conducted in Chinese patients with PAH. The study was held in 18 centers between April 28, 2022, and October 14, 2022. A total of 344 patients were randomly assigned at a 3:1 ratio to the experimental and control groups, respectively, and each group received BoNTA or a BoNTA placebo once. Randomization was performed with an interactive web response system (IWRS) from Almac Clinical Technologies. All investigators, clinical staff involved with the study, and patients were blinded to the treatment assignment throughout the study. The efficacy and safety were evaluated at 1, 4, 8, and 16 weeks after treatment. This study was conducted following the ethical principles of the Declaration of Helsinki, and was first approved by the Ethics Committee at West China Hospital, Sichuan University (no. 2022-21). All the other sites followed thereafter. The signed informed consent was obtained before any study-related procedures.

### Patient Population

The patients (*n* = 344) were between 18 and 65 years old and had persistent bilateral PAH. They had a Hyperhidrosis Disease Severity Scale (HDSS) score of 3 or 4 and a baseline gravimetric measurement of spontaneous resting sweat production of at least 50 mg/axilla, measured over 5 minutes at room temperature.^10^ Patients with secondary hyperhidrosis or a medical condition that could interfere with BoNTA treatment were excluded. Pregnant, lactating, or prospective pregnant female patients also were excluded from the study.

### Botulinum Toxin Type A (BoNTA) and Placebo Treatment

The experimental group received intradermal injection of BoNTA (Hengli, Lanzhou Biotechnique Development Co. Ltd., Lanzhou, Gansu, China). The control group received intradermal injection of BoNTA placebo (Lanzhou Biotechnique Development Co. Ltd., Lanzhou, Gansu, China).

The hyperhidrotic area of each axilla was identified with the Minor's iodine-starch test. One vial (100 U/0 U) of BoNTA or BoNTA placebo was diluted with 2.5 mL of 0.9% sterile normal saline (4 U/0 U per 0.1 mL). A marker pen was used to draw approximately 10 squares with 1- to 2-cm sides in the injection area. After disinfection with iodine, a 30-gauge needle was inserted at a 45-degree angle into the dermis with the bevel facing up, and the approximate depth of each injection was 2 mm. The dose was 5 U/0 U (0.1 mL) at the center of each square, with a total dose of 50 U/0 U (1.0 mL) per axilla. The total dose for both axillae was 100 U/0 U (2.0 mL). The patient remained under observation for 30 minutes after the injection. A single treatment was given, and 16-week follow-up was then conducted. The use of any antiperspirant or deodorant products was prohibited within 48 hours before follow-up visits.

### Endpoints and Assessments

#### Primary Efficacy Endpoint

The proportion of patients who experienced an over 50% reduction in axillary sweat weight (by gravimetric measurement) at Week 4 posttreatment compared to baseline was the primary efficacy endpoint, assessed by the average sweat weight of the bilateral axillae after a 5-minute rest at room temperature.

#### Secondary Efficacy Endpoints

Six secondary efficacy endpoints were assessed.

1. The percentage change in axillary sweat weight (gravimetric measurement) at Weeks 1, 4, 8, and 16 weeks posttreatment compared to baseline;
2. The duration of axillary sweat weight reduction over 50% compared with baseline;
3. The patients' satisfaction scores at Weeks 1, 4, 8, and 16 after treatment; the patients' satisfaction scores were evaluated on a questionnaire with a 9-point scale (Table 1).^11,12^
4. The proportion of patients with an improvement over 2 grades in HDSS scores at Weeks 1, 4, 8, and 16 posttreatment compared to baseline;
5. The changes in the axillary hyperhidrotic area (by Minor's iodine-starch test) at Weeks 4, 8, and 16 posttreatment compared to baseline;
6. The relief rate and overall response rate of axillary bromhidrosis grade at Weeks 1, 4, 8, and 16 posttreatment (evaluated only in patients with baseline bromhidrosis grades of 2 and 3).^13^

**Table 1.** Patients' Assessment of Treatment Satisfaction Scale

Score	Description
+4	Complete abolition of signs and symptoms (nearly 100% improvement)
+3	Substantial improvement (nearly 75% improvement)
+2	Moderate improvement (nearly 50% improvement)
+1	Slight improvement (nearly 25% improvement)
0	No change
−1	Slight worsening (nearly 25% worse)
−2	Moderate worsening (nearly 50% worse)
−3	Substantial worsening (nearly 75% worse)
−4	Very substantial worsening (nearly 100% worse)

The evaluation criteria were as follows: Relieved, no odor in the armpit area (grade 0); significantly improved, mild odor after physical labor or sweating (grade 1); not improved, no significant change between before and after treatment. The relief rate was the number of relieved cases, divided by the total number of cases times 100%. The overall response rate was the number of the relieved cases plus the number of significantly improved cases, divided by the total number of cases times 100%. The total number of cases was the total number of patients with baseline bromhidrosis grades 2 or 3.

#### Safety Assessments

Safety assessments included adverse drug reactions (ADRs), adverse events (AEs), serious adverse events (SAEs), serious adverse drug reactions (SADRs), vital signs, physical examinations, and clinical laboratory measurements. Any AE, SAE, ADR, or SADR observed by the investigator or reported by the patient throughout the study was collected, evaluated, and classified.

### Sample Size

It was assumed that the response rate for the treatment group, defined as over 50% reduction in axillary sweat weight at Week 4 posttreatment compared to baseline, was 60%, and the response rate for the control group was 36%. Considering the α set at 0.05 for 2-sided test and a drop-out rate of 20%, the sample size would be 344 patients (258 in the experimental group and 86 in the control group) with a 3:1 ratio between the 2 groups.

### Statistical Analysis

All statistical analyses were performed with SAS 9.4 Software. Demographic and baseline characteristics as well as efficacy analysis were based on the full analysis set (FAS). The primary and secondary efficacy analyses were primarily based on the evaluable population (EP) set, with supplementary analyses based on the intention to treat population (ITTP) set. Safety analyses were based on the safety set (SS). Continuous variables were described as mean ± SD, and categorical variables were expressed by frequency (percentage, %). The number of responses and response rates were calculated and compared with the chi-square test or Fisher's precision probability test. The difference in response rates between the groups and the 95% CI were calculated with the Wald method. For comparisons of duration, Kaplan–Meier methods were used. The median time and its 95% CI were calculated, and the Kaplan–Meier curves were plotted. The log-rank tests were used for intergroup comparisons. A *P* value of <.05 was considered statistically significant.

## RESULTS

### Patient Disposition and Baseline Characteristics

**Figure 1.**
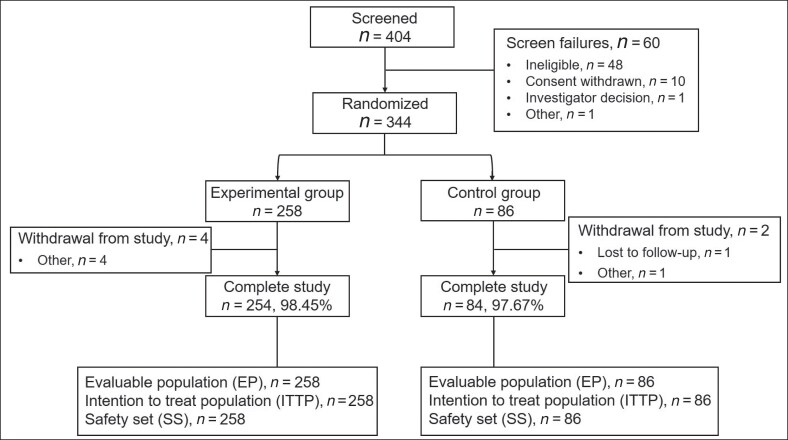
The disposition of the patients.

Overall, 344 patients were randomly assigned to the experimental group (*n* = 258) or the control group (*n* = 86). The age of patients in the experimental group ranged from 18 to 64, with an average age of 32.1 (SD 8.65). The age of patients in the control group ranged from 18 to 63, with an average age of 33.5 (10.75). There were 86 (33.33%) male patients and 172 (66.67%) female patients in the experimental group. In the control group, 30 (34.88%) male patients and 56 (65.12%) female patients were included. Among the 344 patients, 338 (98.26%) completed the study, including 254 (98.45%) in the experimental group and 84 (97.67%) in the control group. The follow-up period ranged from 56 to 174 days, with a median of 112 days. Figure 1 shows the disposition of the patients. All 344 patients were included in the EP, ITTP, and SS population analyses. The 2 groups had similar baseline demographic and disease characteristics (Table 2).

**Table 2.** Patients' Baseline Demographics

Characteristic	Experimental group (*n* = 258)	Control group (*n* = 86)	*P* value
Age, years	32.1 ± 8.65	33.5 ± 10.75	.454
Gender, *n* (%)			
Male	86 (33.33)	30 (34.88)	.792
Female	172 (66.67)	56 (65.12)	
Ethnicity, *n* (%)			
Chinese-Han	241 (93.41)	80 (93.02)	.901
Other	17 (6.59)	6 (6.98)	
BMI, kg/m^2^	22.49 ± 3.48	22.65 ± 3.48	.625
Family history of PAH, *n* (%)			
No	196 (75.97)	62 (72.09)	.472
Yes	62 (24.03)	24 (27.91)	
Baseline HDSS, *n* (%)			
1	0	0	.496
2	0	0	
3	179 (69.38)	63 (73.26)	
4	79 (30.62)	23 (26.74)	
Baseline sweat production per axilla, mg	129.26 ± 72.58	120.20 ± 61.16	.487
Right	140.98 ± 83.79	128.08 ± 67.86	.294
Left	117.54 ± 67.78	112.32 ± 65.15	.492
Baseline axillary hyperhidrotic area, cm^2^	48.81 ± 23.839	47.21 ± 22.838	.682
Right	50.61 ± 25.48	48.36 ± 23.66	.608
Left	47.02 ± 24.46	46.07 ± 24.02	.719
Baseline axillary bromhidrosis grade, *n* (%)			
0	0	0	.597
1	0	0	
2	70 (27.13)	24 (27.91)	
3	23 (8.91)	8 (9.30)	
Past medical history/current medical history, *n* (%)			
No	81 (31.40)	26 (30.23)	.840
Yes	177 (68.60)	60 (69.77)	
Medication history, *n* (%)			
No	201 (77.91)	67 (77.91)	>.999
Yes	57 (22.09)	19 (22.09)	
Nonpharmacological treatment history, *n* (%)			
No	240 (93.02)	83 (96.51)	.242
Yes	18 (6.98)	3 (3.49)	
Surgical history, *n* (%)			
No	216 (83.72)	64 (74.42)	.055
Yes	42 (16.28)	22 (25.58)	

BMI, body mass index; HDSS, hyperhidrosis disease severity scale; PAH, primary axillary hyperhidrosis.

### Efficacy Evaluations

#### Primary Efficacy Assessment

**Figure 2.**
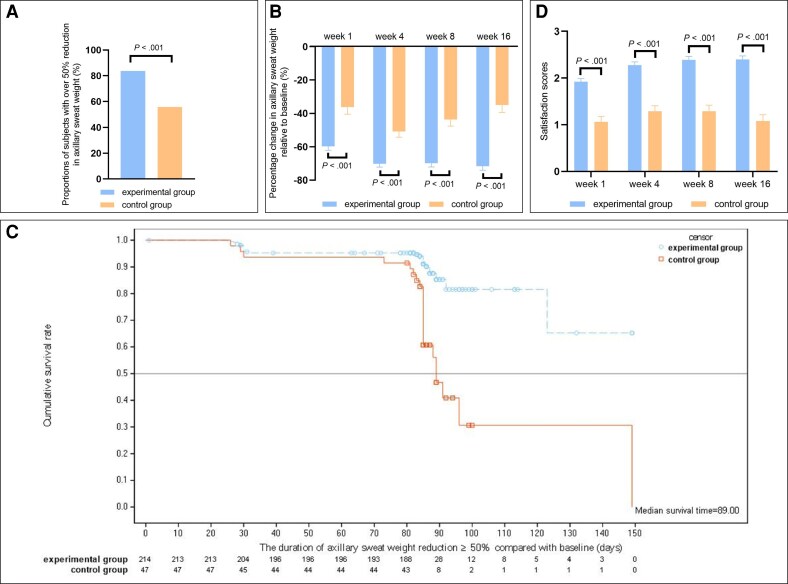
Efficacy evaluations of the primary efficacy endpoint and partial secondary efficacy endpoints. (A) The proportions of patients who experienced over 50% reduction in axillary sweat weight at Week 4 posttreatment compared to baseline; (B) the percentage change in axillary sweat weight relative to baseline at Weeks 1, 4, 8, and 16 posttreatment; (C) the median duration of over 50% reduction in axillary sweat weight compared with baseline; (D) the satisfaction scores measured at Weeks 1, 4, 8, and 16 posttreatment.

According to the EP analysis, the proportions of patients in the experimental group and the control group who experienced over 50% reduction in axillary sweat weight at Week 4 posttreatment compared to baseline were 83.72% (216/258) and 55.81% (48/86), respectively. The between-group difference was 27.91% (95% CI, 16.49-39.33; *P* < .001). The ITTP analysis results were consistent with the EP results (Figure 2A).

### Secondary Efficacy Assessment

**Figure 3.**
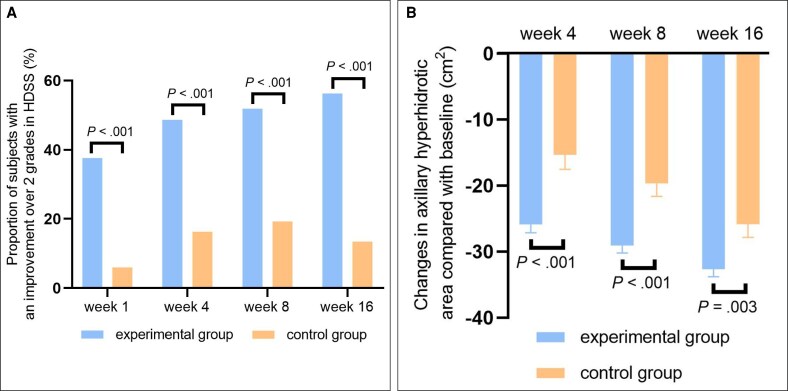
Efficacy evaluations of HDSS scores and axillary hyperhidrotic area. (A) The proportion of patients with an improvement over 2 grades in HDSS scores at Weeks 1, 4, 8, and 16 posttreatment compared to baseline. (B) The changes in the axillary hyperhidrotic area compared with the baseline at 4, 8, and 16 weeks posttreatment. HDSS, hyperhidrosis disease severity scale.

**Figure 4.**
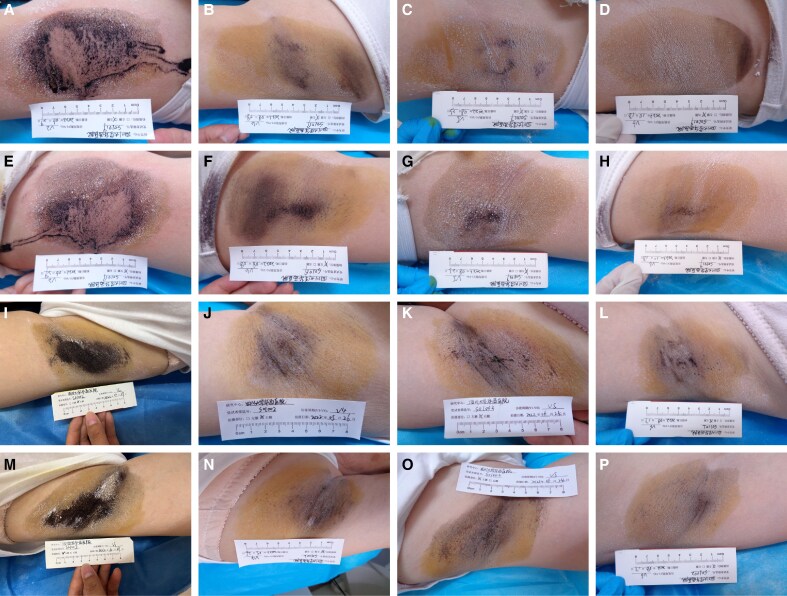
In the treatment group, a 35-year-old female patient underwent intradermal injection of 50 U BoNTA per axilla. (A-H) Baseline and follow-up photographs of axillary hyperhidrotic areas. (A) Right axilla, baseline; (B) right axilla, 4 weeks posttreatment; (C) right axilla, 8 weeks posttreatment; (D) right axilla, 16 weeks posttreatment; (E) left axilla, baseline; (F) left axilla, 4 weeks posttreatment; (G) left axilla, 8 weeks posttreatment; (H) left axilla, 16 weeks posttreatment. In the control group, a 33-year-old female patient underwent intradermal injection of 50 U BoNTA placebo per axilla. (I-P) Baseline and follow-up photographs of axillary hyperhidrotic areas. (I) Right axilla, baseline; (J) right axilla, 4 weeks posttreatment; (K) right axilla, 8 weeks posttreatment; (L) right axilla, 16 weeks posttreatment; (M) left axilla, baseline; (N) left axilla, 4 weeks posttreatment; (O) left axilla, 8 weeks posttreatment; (P) left axilla, 16 weeks posttreatment. BoNTA, botulinum toxin type A.

**Figure 5.**
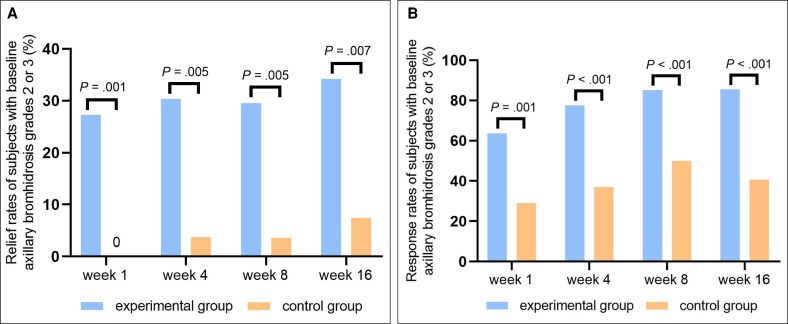
Efficacy evaluations of axillary odor grades. (A) The relief rates at Weeks 1, 4, 8, and 16 posttreatment (patients with baseline axillary odor grades of 2 and 3). (B) The total response rates at Weeks 1, 4, 8, and 16 posttreatment (patients with baseline axillary odor grades of 2 and 3).

1. The EP analysis demonstrated that the percentage change in axillary sweat weight relative to baseline at Weeks 1, 4, 8, and 16 posttreatment in the experimental group was −59.72 ± 2.41%, −70.19 ± 1.98%, −69.82 ± 2.29%, and −71.61 ± 2.48%, respectively. In the control group, the percentage change was −36.22 ± 4.2%, −50.78 ± 3.48%, −43.59 ± 3.98%, and −34.99 ± 4.36%, respectively (*P* < .001, Figure 2B).
2. The EP analysis demonstrated that at the end of the trial, the median duration of over 50% reduction in axillary sweat weight compared with baseline had not yet been reached in the experimental group. The median duration in the control group was 12.57 weeks (*P* < .001, Figure 2C).
3. The EP analysis showed that the satisfaction scores measured at Weeks 1, 4, 8, and 16 posttreatment in the experimental group were 1.92 ± 0.07, 2.28 ± 0.07, 2.39 ± 0.08, and 2.40 ± 0.08, respectively. The satisfaction scores in the control group were 1.06 ± 0.12, 1.29 ± 0.12, 1.29 ± 0.13, and 1.08 ± 0.14, respectively (*P* < .001, Figure 2D).
4. The EP analysis showed that the proportions of patients in the experimental group with an improvement over 2 grades in HDSS scores at Weeks 1, 4, 8, and 16 posttreatment compared to baseline were 37.65% (96/255), 48.61% (122/251), 51.85% (126/243), and 56.28% (121/215), respectively. In the control group, the proportions were 6.02% (5/83), 16.25% (13/80), 19.28% (16/83), and 13.43% (9/67), respectively (*P* < .001, Figure 3A).
5. The EP analysis showed that the changes in the axillary hyperhidrotic area compared with the baseline in the experimental group at 4, 8, and 16 weeks posttreatment were −25.90 ± 1.24 cm^2^, −29.08 ± 1.13 cm^2^, and −32.69 ± 1.13 cm^2^, respectively. In the control group, the changes were −15.31 ± 2.22 cm^2^, −19.66 ± 1.96 cm^2^, and −25.85 ± 2.01 cm^2^, respectively. The *P* values were *P* < .001, *P* < .001, and *P* = .003, respectively (Figures 3B, 4).
6. The EP analysis demonstrated that in the patients with baseline axillary bromhidrosis grades of 2 and 3, the relief rates in the experimental group at Weeks 1, 4, 8, and 16 posttreatment were 27.27% (24/88), 30.34% (27/89), 29.55% (26/88), and 34.21% (26/76), respectively. The relief rates in the control group were 0%, 3.70% (1/27), 3.57% (1/28), and 7.41% (2/27), respectively. The *P* values were *P* = .001, *P* = .005, *P* = .005, and *P* = .007, respectively (Figure 5A).

The EP analysis demonstrated that in the patients with baseline axillary bromhidrosis grades of 2 and 3, the total response rates in the experimental group at Weeks 1, 4, 8, and 16 posttreatment were 63.64% (56/88), 77.53% (69/89), 85.23% (75/88), and 85.53% (65/76), respectively. The total response rates in the control group were 29.03% (9/31), 37.04% (10/27), 50.00% (14/28), and 40.74% (11/27), respectively. The *P* values were *P* = .001, *P* < .001, *P* < .001, and *P* < .001, respectively (Figure 5B).

The ITTP analysis results of the secondary efficacy results were consistent with the EP results.

### Safety Evaluations

In the experimental group, 202 (78.29%) patients experienced AEs, 3 (1.16%) patients experienced SAEs, and 8 (3.10%) patients experienced grade 3 or higher AEs. In the control group, 59 (68.60%) patients experienced AEs, 2 (2.33%) patients experienced SAEs, and 2 (2.33%) patients experienced grade 3 or higher AEs.

In total, 165 cases and 181 occurrences of ADRs were observed in the experimental group, with an incidence of 63.95%. One hundred and sixty-three (63.18%) patients experienced grade 1 ADRs, and 2 (0.78%) patients experienced grade 2 ADRs.

In total, 49 cases and 56 occurrences of ADRs were observed in the control group, with an incidence of 56.98%. All ADRs were grade 1. Neither group experienced SADRs nor grade 3 or higher ADRs (Table 3).

**Table 3.** Summary of Safety Evaluations, SS Population

	Experimental group (*n* = 258)		Control group (*n* = 86)		*P* value
	*n* (%)	Occurrence	*n* (%)	Occurrence	
AEs	202 (78.29)	405	59 (68.60)	116	.069
ADRs	165 (63.95)	181	49 (56.98)	56	.248
SAEs	3 (1.16)	3	2 (2.33)	2	.602
SADRs	0	0	0	0	—
AEs, grade 3 or higher	8 (3.10)	9	2 (2.33)	3	>.999
ADRs, grade 3 or higher	0	0	0	0	—
AEs leading to withdrawal	0	0	0	0	—
ADRs leading to withdrawal	0	0	0	0	—
SUSAR	0	0	0	0	—
AEs leading to death	0	0	0	0	—
ADRs leading to death	0	0	0	0	—

ADR, adverse drug reaction; AE, adverse event; SADR, serious adverse drug reaction; SAE, serious adverse event; SS, safety set; SUSAR, suspected serious adverse drug reaction.

ADRs with an incidence higher than 1% in the experimental group included injection site pain and injection site itching. ADRs with an incidence higher than 1% in the control group included injection site pain, itching, swelling, headache, dizziness, muscle pain, and abnormal skin odor. The outcome of 181 ADRs in the experimental group and 56 ADRs in the control group was recovery/healing. Neither group experienced suspected unexpected serious adverse reactions (SUSARs) nor AEs/ADRs leading to withdrawal or death.

## DISCUSSION

This study was the first Phase 3, multicenter, randomized, double-blind, placebo-controlled study of BoNTA treatment for PAH in Chinese patients. Both quantitative and qualitative methods were employed to assess the efficacy.^14^

Although alternative treatment modalities for PAH have been emerging in recent years, intradermal toxin injections remain a preferred option. Intradermal BoNTA injections had better efficacy than fractional microneedle radiofrequency for PAH, as illustrated by HDSS, dermatology life quality index (DLQI), and participants' satisfaction scores.^15^ BoNTA treatment is less painful, less expensive, and needs a smaller number of sessions.^16^ Of note, the duration of efficacy increases with repeated BoNTA injections in patients with PAH. The results of an Australian cohort study confirmed high patient satisfaction with onabotulinumtoxin A treatment for PAH and enhanced durability with repeated treatments, and offered hope to those who have previously failed other modalities.^9,17-19^ Another potential benefit might be the improvement of hyperhidrosis of other sites following the BoNTA treatment of PAH.^20^

According to our study, the experimental group outperformed the control group on both the primary endpoint and secondary endpoints. Patients in the experimental group experienced rapid onset of action, with significant reduction in axillary sweat weight starting 1 week after treatment. The duration of efficacy was significantly longer for the experimental group than for the control group, and patients' satisfaction was higher as well. Starting from Week 4 posttreatment, the response rate for patients with axillary bromhidrosis in the experimental group was approximately 80%, compared to less than 50% in the control group. The relief rate for bromhidrosis in the experimental group was approximately 30% through the study, compared to less than 10% in the control group. These results suggested that BoNTA could serve as a treatment option for PAH cases with axillary bromhidrosis. The patients' satisfaction scores, improvements in HDSS scores, reduction in axillary hyperhidrotic area, and relief rates for cases with axillary bromhidrosis in the experimental group were consistent with the sustained reduction in axillary sweat weight through the study.

The primary endpoint results of the current study did not fully align with those reported. Noticeably, the control group had a response rate of 55.81% with the placebo treatment, which was considerably high. According to the clinical trials with similar design, the control group had a response rate ranging from 34.7% to 36%.^11,21^ We speculated that the relatively high response rate in the control group in our study might have 2 reasons. First, the previous similar studies focused on European PAH patients, whereas the present study recruited Chinese patients. Differences in ethnicity may have influenced the clinical outcomes. Second, primary hyperhidrosis is an autonomic nervous system–related disorder. The response rate in the control group might be impacted by psychological effects. On the secondary efficacy results, the treatment group still demonstrated superior outcomes across multiple efficacy endpoints.

Although a rigorous quantitative primary endpoint was utilized to measure efficacy, its clinical application is limited. Notably, the qualitative between-group variance (for instance, difference in patients' satisfaction and HDSS scores) seemed to be more prominent than the quantitative one (for instance, difference in axillary sweat weight and axillary hyperhidrotic area). A reasonable therapeutic goal for treating PAH is to reduce sweating to a level that minimizes interference with daily activities, as indicated by an HDSS score of 1 or 2.^10^ The Canadian hyperhidrosis advisory committee defined treatment success by an HDSS score change from a 4 or 3 to 2 or 1, or a score change from 2 to 1. Treatment failure was defined as lack of improvement in HDSS score after 1 month of treatment or intolerable therapy. It has been shown that a 1-point improvement in the HDSS score correlates with a 50% reduction in sweating. An improvement of 2 points corresponds to an 80% sweat reduction.^2^ Gravimetric measurements and the Minor's iodine-starch test are rarely performed in clinical settings. However, they can help determine HH severity, guide treatment options, and gather data for research.

The dose of 50 U per axilla was determined based upon the clinical experience in our country. The toxin dose varies largely in the previous trials, and the efficacy of single BoNTA treatment with different doses for PAH varies largely as well.^7,22,23^ The available data seemed to argue for a markedly prolonged efficacy of “high-dose” BoNTA. No significant difference in treatment efficacy was apparent between the 75-U and 50-U BoNTA doses per axilla. However, some patients having larger axillary sweating surface areas may benefit from a 75-U dose, because the number of injection sites can be increased.^10^ A major disadvantage of a relatively low dose is the reappearance of clinically relevant excessive sweating, which usually occurs within 6 months. Repeated applications may induce neutralizing antibodies, so the optimal dose should be administered and repeated applications should be limited.

Overall, the BoNTA treatment was well tolerated. The 2 groups had comparable rates of AEs and ADRs, and the severity was mostly grade 1 or 2. The most common ADR was pain related to the injection procedure, which could be alleviated by tailoring number of injections, applying liposomal lidocaine, cooling with frozen gel packs, or toxin dilution with lidocaine.^24-27^

Although the BoNTA treatment is effective and minimally invasive, it requires multiple repeated sessions. Alternatively, thoracic sympathectomy provides long-term efficacy for PAH. The primary complication of sympathectomy is compensatory hyperhidrosis, which can be managed with low-dose BoNTA.^28^ Studies comparing BoNTA and sympathectomy for treating axillary or palmar hyperhidrosis have yielded inconsistent conclusions regarding the treatment option selection or preference.^29-32^ Practitioners need to make decisions based on each patient's specific circumstances.

One major limitation of this study is that only 1 BoNTA treatment was evaluated. In general, botulinum toxin A (BoNT-A) injections for the treatment of PHH need to be repeated 2 to 3 times per year to maintain a therapeutic effect. Second, the study evaluated only 1 toxin dose. The optimal dose effectively reduces sweating to normal physiological levels for as long as possible while minimizing side effects. Although 50 U of BoNTA per axilla was safe and effective in this trial population, the results may not be generalizable to the other ethnic populations. Some patients also may need a dose adjustment to achieve optimal clinical results. Third, the follow-up period was limited to 16 weeks, and follow-up studies with longer observation periods are necessary to confirm sustainability and long-term effects.

## CONCLUSIONS

In conclusion, Chinese patients with PAH who received 1 intradermal 50-U BoNTA treatment experienced a significant reduction in axillary sweat weight, axillary hyperhidrotic area, HDSS score, and axillary bromhidrosis grade. The therapeutic effect was maintained for 16 weeks posttreatment, with high satisfaction and a favorable safety profile.
